# A Fraction of CD8+ T Cells from Colorectal Liver Metastases Preferentially Repopulate Autologous Patient-Derived Xenograft Tumors as Tissue-Resident Memory T Cells

**DOI:** 10.3390/cancers14122882

**Published:** 2022-06-10

**Authors:** Frank Liang, Lisa M. Nilsson, Fabian Byvald, Azar Rezapour, Helena Taflin, Jonas A. Nilsson, Ulf Yrlid

**Affiliations:** 1 Department of Microbiology and Immunology, Institute of Biomedicine, Sahlgrenska Academy, University of Gothenburg, 40530 Gothenburg, Sweden; gusbyvfa@student.gu.se (F.B.); azar.rezapour@gu.se (A.R.); 2 Sahlgrenska Translational Melanoma Group, Sahlgrenska Center for Cancer Research, Department of Surgery, Institute of Clinical Sciences, University of Gothenburg, 40530 Gothenburg, Sweden; lisa.m.nilsson@surgery.gu.se (L.M.N.); jonas.a.nilsson@surgery.gu.se (J.A.N.); 3 Harry Perkins Institute of Medical Research, UWA Centre for Medical Research, University of Western Australia, Nedlands, WA 6009, Australia; 4 Department of Surgery, Institute of Clinical Sciences, Sahlgrenska University Hospital, University of Gothenburg, 41345 Gothenburg, Sweden; helena.taflin@vgregion.se

**Keywords:** colorectal liver metastases, tumor-infiltrating lymphocytes, tissue-resident memory T cells, patient-derived xenograft model, MEK inhibition

## Abstract

**Simple Summary:**

Treatment options for colorectal cancer (CRC) patients with liver metastases are often limited to liver surgery with or without chemotherapy. However, not all patients present operable colorectal liver metastases (CRLMs). Thus, alternative therapies that exploit the anti-tumor potential of tumor-infiltrating lymphocytes (TILs) are being evaluated. The establishment of markers connecting the phenotype to the function of tumor-reactive CD8+ TILs could aid diagnostic and therapeutic advances. In this regard, tissue-resident memory T cells (T_RM_ cells) could be a potential candidate for therapies targeting TILs. Putative tumor-reactive T_RM_ cells among CD8+ TILs likely co-express CD103 and CD39, since these markers indicate stable tumor residency and repeated response to antigens from the tumor environment, respectively. Our phenotypic and functional analyses of TILs in CRLM, with a specific focus on CD103+CD8+ T_RM_ cells, may guide the improvement of TIL-mediated CRC treatments.

**Abstract:**

The diversity of T cells in the human liver may reflect the composition of TILs in CRLM. Our ex vivo characterization of CRLM vs. adjacent liver tissue detected CD103+CD39+CD8+ T_RM_ cells predominantly in CRLM, which prompted further assessments. These T_RM_ cells responded to cognate antigens in vitro. As functional activities of autologous TILs are central to the implementation of personalized cancer treatments, we applied a patient-derived xenograft (PDX) model to monitor TILs’ capacity to control CRLM-derived tumors in vivo. We established PDX mice with CRLMs from two patients, and in vitro expansion of their respective TILs resulted in opposing CD4+ vs. CD8+ TIL ratios. These CRLMs also displayed mutated *KRAS*, which enabled trametinib-mediated inhibition of MEK. Regardless of the TIL subset ratio, persistent or transient control of CRLM-derived tumors of limited size by the transferred TILs was observed only after trametinib treatment. Of note, a portion of transferred TILs was observed as CD103+CD8+ T_RM_ cells that strictly accumulated within the autologous CRLM-derived tumor rather than in the spleen or blood. Thus, the predominance of CD103+CD39+CD8+ T_RM_ cells in CRLM relative to the adjacent liver and the propensity of CD103+CD8+ T_RM_ cells to repopulate the autologous tumor may identify these TILs as strategic targets for therapies against advanced CRC.

## 1. Introduction

A high frequency of patients with CRC (25–50%) eventually present with metastasized tumors, typically in the liver [1], and CRC remains the second most common cause of cancer-related mortality [2]. In primary CRC, the specific phenotype, location and frequency of intratumoral T cells, commonly referred to as TILs, are vital parameters in the prognosis [3,4]. Moreover, multiple reports also associate a favorable prognosis with CD8+ TIL levels in CRLM [5,6,7], which again emphasizes the role of TILs in anti-tumor responses.

The liver contains several immune cells with phenotypes, functions and frequencies that are distinctive for this highly metabolic tissue [8,9,10]. However, simultaneous analyses of antigen-presenting cells (APCs) and T cells in human CRLM vs. autologous liver tissue are generally rare. As a high level of CD8+ TILs is favorable in CRC, identification of tumor-specific TILs has been pursued to potentially refine the prognosis. In this regard, putative CD103+CD39+CD8+ tumor-reactive TILs in primary tumors have previously been assessed by us and others [11,12,13]. These CD8+ TILs express CD103 (integrin alpha E), which is involved in tissue retention, and upregulate CD39 (ectonucleotidase) due to, e.g., continuous activation, which suggests that they exert their effector functions while steadily residing in the tumor as T_RM_ cells. The human liver hosts T_RM_ cells poised to eliminate cognate pathogens [10], but whether potentially tumor-specific T_RM_ cells infiltrate CRLMs and the levels at which T_RM_ cells are found in CRLM or the adjacent liver are largely unknown.

With regard to clinical outcome, high numbers of intratumoral CD8+ T_RM_ cells have been correlated with improved survival in multiple cancers [14,15], including primary CRC [16]. Hence, intratumoral accumulation of T_RM_ cells may represent the initial steps towards tumor elimination. However, varying degrees of inflammation in the tumor environment likely drive general immune cell infiltration rather than the selective recruitment of T_RM_ cells. Intriguingly, various mouse tumor models showed a dramatic increase in CD8+ TILs after treatment with only trametinib, an inhibitor of mitogen-activated protein kinase kinase (MEK) of the RAS pathway, or in combination with immunotherapy [17,18,19,20,21]. Although a phase I trial with trametinib monotherapy showed low efficacy in CRC patients [22], MEK inhibition (MEKi) remains relevant in combined therapies targeting, e.g., CRC. Of note, MEKi plus other treatment modalities induced efficient tumor rejection in mice [17,18,19,20,21]. Whether MEKi increases intratumoral T_RM_ cells in patients with CRLM has yet to be determined.

Here, we compared the frequencies and activation states of CD103+CD8+ T_RM_ cells and other conventional T cells, unconventional T cells and APCs in CRLM and the adjacent liver tissue from patients with CRC. Subsequently, we utilized the PDX model and in vitro expansion of TILs, as described in [23], to address whether subcutaneous CRLM-derived tumors in lymphocyte-deficient mice with constitutive human IL-2 expression showed the accumulation of CD8+ T_RM_ cells during MEKi. The obtained data hold promise for the design of treatments targeting CD8+ T_RM_ cells for patients with advanced CRC, particularly those with inoperable liver metastases.

## 2. Results

### 2.1. T Cell Subsets from CRLM and Adjacent Liver Differ in Frequency and Activation Status

**Figure 1:**
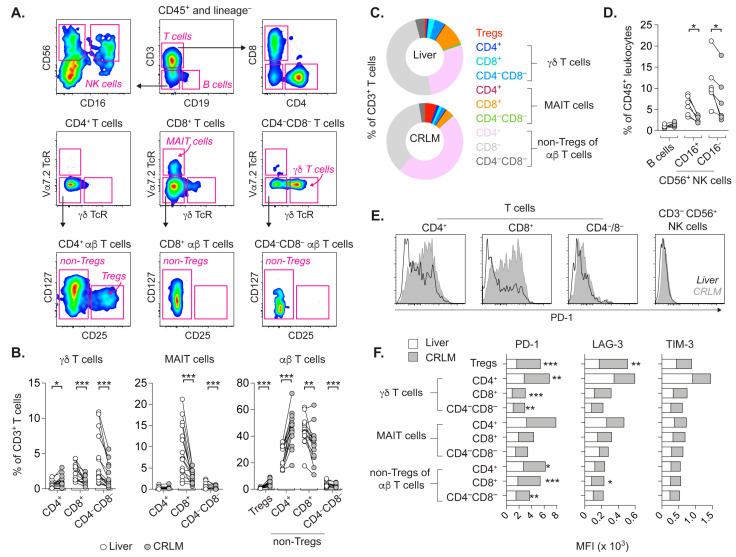
Characterization of lymphocytes in CRLM and adjacent liver tissue. (**A**) Gating strategy of lymphocyte subsets, as shown with single-cell suspension of CRLM. Viable, CD45+ and lineage (CD11c, CD15, CD123)− cells were divided into CD3+ T cells and CD19+ B cells. CD56+ NK cell subsets according to CD16 expression were gated from CD3−CD19− cells. CD3+ T cells were separated into CD4+, CD8+ or CD4−CD8− T cells. Unconventional Vα7.2 TcR+ MAIT cells were mainly among CD8+ T cells, and CD4−CD8− T cells contained γδ T cells expressing γδ TcR. Remaining T cells represent conventional αβ T cells, and CD127dim/−CD25+ Tregs were gated from CD4+ αβ T cells. The CD25− αβ T cells are referred to as non-Tregs. Compiled data on ex vivo percentages (**B**) and proportions (**C**) of indicated T cell subsets among CD3+ T cells in CRLM and adjacent liver. (**D**) Frequencies of B cells and NK cell subsets within CD45+ leukocytes. (**E**) Overlay of histograms on PD-1 staining intensity on T cells and NK cells in liver vs. CRLM. (**F**) Stacked bars show proportion of the mean fluorescence intensity (MFI) for the staining of PD-1, LAG-3 or TIM-3 on T cell subsets in CRLM and liver, respectively (* *p* < 0.05, ** *p* < 0.01, *** *p* < 0.001, Wilcoxon test).

A plethora of immune cell subsets exist in the liver, and CRLMs are likely infiltrated by leukocytes from the blood and surrounding liver tissue. We determined the frequencies of immune cells in CRLM and adjacent macroscopically tumor-free liver tissue from patients with synchronous or metachronous CRC (Supplementary Table S1). Multiple lymphocyte subsets were defined (Figure 1A), including unconventional γδ T cells and mucosal-associated invariant T cells (MAIT cells). These unconventional T cells are normally involved in anti-microbial responses but have also been associated with pro- or anti-tumor responses [24,25,26]. The expression of the γδ T cell receptor (TcR) and Vα7.2 TcR defined γδ T cells and MAIT cells, respectively. Whereas γδ T cells were mainly within CD4−CD8− T cells, MAIT cells were observed predominantly among CD8 T cells.

The absence of γδ TcR and Vα7.2 TcR expression specified conventional T cells, referred to as αβ T cells. As previously reported, CD4+ αβ T cells contained CD127 dim/−CD25+ regulatory T cells (Tregs) [24], and CD25− T cells were denoted as non-Tregs.

Relative to corresponding T cells in the liver, the frequencies and proportions of the more abundant subsets of γδ T cells and MAIT cells among total CD3+ TILs were decreased in CRLM (Figure 1B,C). Regarding αβ T cells in CRLM, Tregs and CD4+ non-Tregs were increased compared to the liver, but CD8+ or CD4−CD8− non-Tregs were decreased (Figure 1B,C). Interestingly, B cells were scarce in both the liver and CRLM, and the frequency of NK cell subsets was lower in CRLM (Figure 1A,D).

The different frequencies of several lymphocyte subsets in CRLM vs. liver could be related to distinct tissue environments. Thus, we assessed the phenotypic T cell activation in these tissues by their expression of programmed death-1 (PD-1), lymphocyte activation gene 3 (LAG-3) and T cell immunoglobulin and mucin domain-containing protein 3 (TIM-3). The mean fluorescence intensity (MFI) describes the expression level of assessed markers. In contrast to T cells, NK cells in the liver or CRLM did not express PD-1 (Figure 1E).

Relative to the liver, several corresponding T cell subsets in CRLM showed significantly higher expression of PD-1 and LAG-3, but not TIM-3 (Figure 1F). Altogether, the contrasting environments of CRLM and the liver are likely represented by differences in the activation states and frequencies of T cell subsets.

### 2.2. CD8+CD103+ T_RM_ Cells from CRLM and Liver Proliferate upon Cognate Stimulation

**Figure 2:**
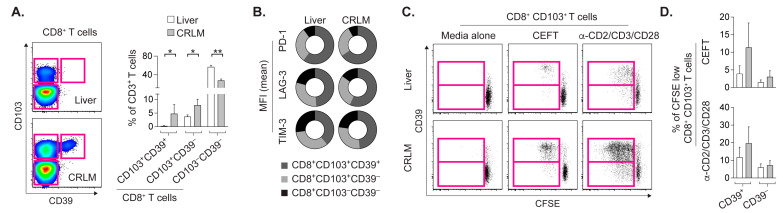
Proliferating CD8+ T_RM_ cells during cognate stimulation express CD39. (**A**) Gating of CD8+ T_RM_ cells expressing CD103 (tissue residency marker) with or without co-expression of CD39 (indicator of extended activation), and ex vivo frequency of T_RM_ cell subsets, plus CD103−CD39−CD8+ T cells among CD3+ T cells in liver and CRLM. (**B**) Proportions of the mean MFI of PD-1, LAG-3 and TIM-3 staining on indicated T cell subsets. (**C**) CFSE-labeled CD8+CD103+T_RM_ cells diluting CFSE dye for 4 days as they proliferate during cognate response to peptide mixture based on microbial antigens (CEFT). Polyclonal stimulation with anti-CD2/CD3/CD28 antibodies on microbeads served as positive control. (**D**) Background-subtracted frequency of the CD8+CD103+ T_RM_ cells expressing CD39 or not among proliferating (CFSE-low) T_RM_ cells. Means with SDs shown (* *p* < 0.05, ** *p* < 0.01, Wilcoxon test).

Since CD103+CD8+ T_RM_ cells in primary CRC correlated with a favorable prognosis [16], and CD103+CD39+CD8+ TILs have been described as tumor-reactive in various solid tumors [11,12], we assessed T cells by detecting these markers. CD103+CD8+ T_RM_ cells were more abundant in CRLM relative to the adjacent liver (Figure 2A). Interestingly, CD103+CD39+CD8+ T_RM_ cells were predominantly found in CRLM.

In both the liver and CRLM, CD103+CD39+CD8+ T_RM_ cells had proportionally higher PD-1, LAG-3 and TIM-3 expression (Figure 2B), which suggests that these T_RM_ cells were exposed to extended stimulation.

To model cognate stimulation of CD103+CD8+ T_RM_ cells in vitro, cells in the liver and CRLM suspensions were labeled with CFSE dye and cultured with peptides based on microbial antigens (Figure 2C). Proliferating CEFT-specific CD103+CD8+ T_RM_ cells, as visualized by their dilution of CFSE dye (i.e., CFSE-low cells), appeared more prominent in CRLM than in the liver. Regardless of the tissue site, most proliferating CD103+CD8+ T_RM_ cells co-expressed CD39 during antigen-specific or polyclonal stimulation (Figure 2D). Whether CD39 levels on CFSE-low T_RM_ cells represent retained or upregulated expression remains undetermined. Nonetheless, CRLM had a higher frequency of CD103+CD39+CD8+ T_RM_ cells capable of cognate responses relative to the adjacent liver.

### 2.3. Prominent DC Activation in CRLM Compared to the Adjacent Liver

CEFT peptides were likely presented to T cells in CRLM and liver suspensions by co-residing APCs. CD163+CD14+ macrophages (MPs) were abundant in both CRLM and the liver (Supplementary Figure S1A,B), and as MPs are heterogeneous, some of the CD163+CD14+ MPs may represent Kupffer cells (liver macrophages). CD163– APCs comprised CD14–CD16– dendritic cells (DCs), plus monocytes (MOs) or MPs that co-expressed CD14 and CD16 or singly expressed these markers. With regard to DCs, we assessed the frequencies of CD123+ plasmacytoid DCs (PDCs) and CD11c+ myeloid DCs (MDCs), as well as the CD141+ vs. CD1c+ subsets of MDCs. Except for PDCs, the frequencies of the other APC subsets were not significantly different in CRLM vs. liver (Supplementary Figure S1B). The PDCs in CRLM had higher HLA-DR (MHC-II) expression compared to liver PDCs. The MDCs in CRLM exhibited higher levels of HLA-DR, T cell co-stimulatory CD80 and co-inhibitory PD-L1 compared to liver tissue (Supplementary Figure S1C). Therefore, CEFT-specific T_RM_ cells in CRLM were likely primarily regulated by co-residing MDCs during antigen presentation.

### 2.4. In-Vitro-Expanded TILs Largely Consist of αβ T Cells Capable of IFN-γ Production

The presence of CD103+CD39+CD8+ T_RM_ cells responding to cognate antigens in CRLM (Figure 2C,D) prompted the expansion of TILs, which may increase the number of scarce tumor-reactive TILs, and enabled us to address whether expanded TILs would accumulate in the autologous tumor as T_RM_ cells to control tumor growth.

**Figure 3:**
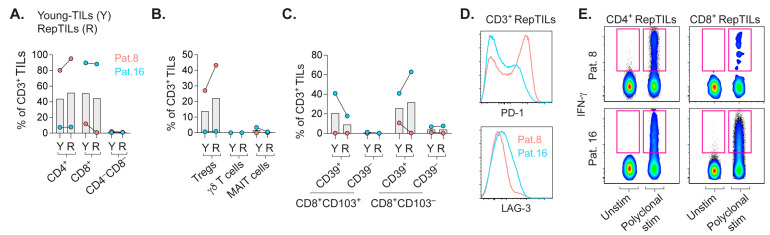
Phenotypic and functional assessment of in vitro–expanded TILs. (**A**) Frequency of CD4+, CD8+ or CD4−CD8− TILs among CD3+ young-TILs or RepTILs from patients 8 and 16, respectively. Frequency of Tregs and unconventional TILs (**B**), plus CD8+CD103+ T_RM_ cells and CD8+CD103− TILs expressing CD39 or not (**C**). (**D**) Histograms of PD-1 and LAG-3 on total RepTILs. (**E**) IFN-γ expression by RepTILs after polyclonal stimulation with microbeads loaded with anti-CD2/CD3/CD28 antibodies or cultured in medium alone (unstim). Bars show the mean.

We expanded TILs in vitro from the CRLM from two patients (Figure 3A–E) for subsequent transfer to lymphocyte-deficient PDX mice bearing subcutaneous tumor implants derived from the autologous CRLM, as previously described [23,27].

Whereas young-TILs refer to TILs expanded as they emigrate from pieces of CRLM, RepTILs consist of young-TILs that were further expanded prior to adoptive transfer. Although frequencies of CD4+ or CD8+ TILs were quite similar between young-TILs and RepTILs, the ratios of these subsets in the respective patients were in stark contrast (Figure 3A). Expanded TILs from patient 8 with a higher frequency of CD4+ TILs also contained more Tregs (Figure 3B). Interestingly, unconventional TILs were not expanded. Regarding T_RM_ cells, expanded TILs from patient 16 that comprised more CD8+ TILs also contained more CD8+CD103+ T_RM_ cells (Figure 3C). The RepTILs from the two patients also expressed contrasting levels of PD-1 and LAG-3 (Figure 3D). Importantly, RepTILs showed a general ability to mount effector responses, as they readily produced IFN-γ upon polyclonal stimulation (Figure 3E).

### 2.5. MEKi after Transfer of RepTILs Modestly Control Growth of Tumor of Limited Size

With the prospect of studying the anti-tumor responses of transferred RepTILs in PDX mice carrying autologous tumor implants, we concluded that this in vivo setting would also offer an apt opportunity to assess the effects of MEKi on TIL responses. The CRLMs from both patients presented a single nucleotide mutation in *KRAS* (Supplementary Figure S2A), which suggested susceptibility to trametinib-mediated MEKi. In line with a phase I trial reporting stable CRC disease after trametinib monotherapy [22], relatively stable tumor volume was observed in trametinib-treated PDX mice with the tumor implant from patient 8 (Supplementary Figure S2B).

To evaluate the anti-tumor capacity of in vitro–expanded TILs with contrasting CD4+ vs. CD8+ TIL ratios, RepTILs were transferred to tumor-implant-bearing PDX mice with constitutive human IL-2 expression to maintain adequate TIL levels in vivo. Qualitative analysis of TIL responses consisted of the transfer of RepTILs once tumor implants surpassed or remained below arbitrarily chosen volumes. Despite the opposing TIL subset ratios between the two patients, RepTILs from neither patient were more efficient than the other in controlling tumor growth (Supplementary Figure S2C). Tumor control was not enhanced when RepTILs were transferred, while tumor implants were of smaller size.

However, PDX mice with smaller tumor implants derived from patient 8 showed relatively persistent tumor control after the transfer of RepTILs and trametinib treatment around 3 weeks later, but this effect was transient for PDX mice representing patient 16 (Supplementary Figure S2D).

### 2.6. CD8+ T_RM_ Cells Prefer to Repopulate the Autologous CRLM-Derived Tumor Implant

**Figure 4:**
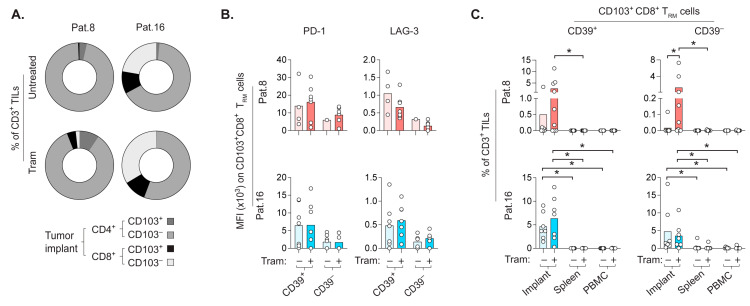
Portion of transferred RepTILs accumulates in the tumor implant as CD8+ T_RM_ cells. PDX mice with subcutaneous tumor implants derived from CRLM of patient 8 or 16 received autologous RepTILs with or without trametinib treatment, initiated around 3 weeks (22 ± 3 days) later. (**A**) Proportions of CD103+ and CD103− subsets of CD4+TILs and CD8+ TILs in tumor implants of untreated (RepTIL transfer alone) and treated (trametinib after RepTIL transfer) PDX mice, based on their frequencies within transferred CD3+ RepTILs. (**B**) MFI of PD-1 and LAG-3 staining on CD103+CD8+ T_RM_ cell subsets in tumor explants. (**C**) Frequency of CD103+ CD8+ T_RM_ cell subsets among transferred CD3+ RepTILs in tumor implants, as well as spleens and PBMCs of PDX mice from indicated groups. Bars show the mean. Transparent and opaque bars indicate untreated and trametinib-treated groups, respectively, for patient 8 (red) and patient 16 (blue). (* *p* < 0.05, Wilcoxon test and Mann–Whitney test for paired and unpaired comparisons, respectively).

Trametinib mediates a remarkable increase in CD8+ T cells within various mouse tumors [17,18,19,20,21]. Although the transfer of RepTILs and subsequent trametinib treatment induced modest control of smaller CRLM-derived tumors, we utilized our PDX model to address the fate of transferred RepTILs, particularly whether trametinib increased their presence in the autologous tumor as activated CD8+ T_RM_ cells (Figure 4A–C).

The TILs in tumor implants were essentially CD45RO+ memory T cells, regardless of trametinib treatment (Supplementary Figure S3A–C). Whereas memory CD8 T cells in PDX tumors from both patients comprised a higher frequency of CD103+CD39+CD8+ T_RM_ cells, CD103–CD39– memory CD8 T cells were prominent in the tumor implant from patient 16. The highly contrasting ratio of in vitro–expanded CD4+ vs. CD8+ RepTILs between the two patients was less apparent in vivo, as tumor implants from PDX mice representing patient 16 also had a large proportion of CD4+ TILs (Figure 4A). Although tumor implants of patient 16 still contained a larger fraction of CD8+ TILs, CD103−CD4+ TILs were proportionally dominant in PDX mice for both patients. These CD4+ TILs contained Tregs, and overall, their frequency and expression of LAG-3 or PD-1 were comparable between the untreated and trametinib-treated PDX mice (Supplementary Figure S4A,B).

The CD103+CD39+CD8+ T_RM_ cells in tumor implants tended to have higher PD-1 and LAG-3 expression than CD103+CD39−CD8+ T_RM_ cells (Figure 4B), suggesting the possibility of distinguishing the activation state of T_RM_ cells via CD39 expression.

Despite a low frequency of CD8+ RepTILs (Figure 3A), the tumor implant of patient 8 had a more dramatic fold increase in CD103+CD8+ T_RM_ cells after trametinib treatment than untreated PDX mice (Figure 4C). In general, the increase in CD103+CD8+ T_RM_ cells in tumor implants from patient 8 appeared to be related to trametinib treatment. However, the frequencies of intratumoral CD103+CD8+ T_RM_ cells in treated and untreated PDX mice for patient 16 were similar. Interestingly, CD103+CD8+ T_RM_ cells preferred to accumulate in the tumor implant derived from both patients, rather than in the spleen or circulation of the PDX mice.

### 2.7. CD8+ T_RM_ Cells Display Strategic Intratumoral Positioning after Trametinib Treatment

**Figure 5:**
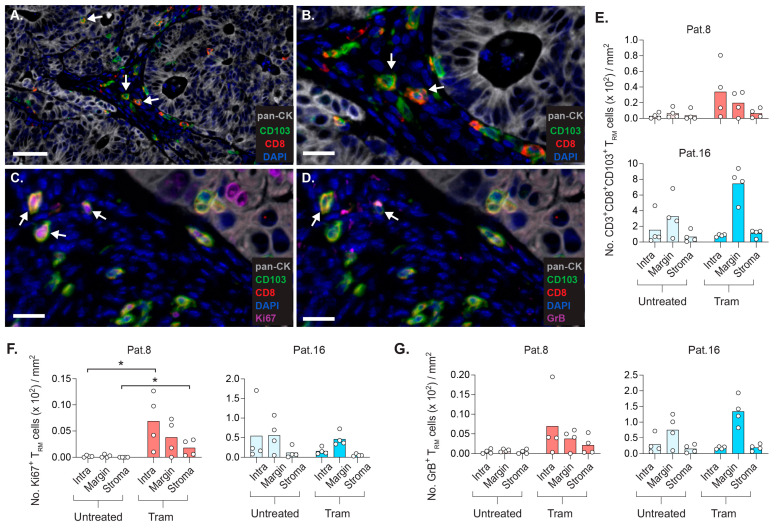
Trametinib promotes increased density of CD8+ T_RM_ cells at specific sites of tumor implant. Tissue staining of CD3+CD103+CD8+ T_RM_ cells and the expression of Ki67 (proliferation marker) and Granzyme B (GrB, involved in T-cell-mediated killing of target cells) in tumor implants of patients 8 and 16. PDX mice were treated as described in Figure 4. (**A**) Pan-cytokeratin (pan-CK)+ tumor epithelia and pan-CK- tumor stroma contained CD3+ TILs with variable CD8 and CD103 expression. Individual cells are defined by DAPI+ cell nuclei. Arrows denote CD8+CD103+ T_RM_ cells in stromal and intraepithelial tumor compartments. The arrows indicate T_RM_ cells (**B**) and examples of CD8+ T_RM_ cells expressing Ki67 (**C**) or GrB (**D**). (**E**) Density of CD8+CD103+ T_RM_ cells within tumor epithelia (intra) and at the tumor margin (0–49 μm away from tumor bed) or stroma (50–100 μm away from tumor bed). Density of CD8+CD103+ T_RM_ cells expressing Ki67 (**F**) or GrB (**G**). Scale bars: 50 μm in A, and 20 μm in B-D. (* *p* < 0.05, Mann–Whitney test).

The explicit accumulation of CD103+CD8+ T_RM_ cells in the tumor implant prompted the analysis of their location relative to the tumor epithelia and stroma (Figure 5A–D).

The staining of pan-cytokeratin (pan-CK) enabled the separation of pan-CK+ tumor epithelia and pan-CK– stroma. The stromal CD3+ TILs comprised CD103+CD8+ T_RM_ cells and CD103−CD8+ TILs, as well as CD8− TILs that represent CD4 TILs with or without CD103 expression (Figure 5A,B). Intraepithelial CD103+CD8+ T_RM_ cells were also detected. The reason for the increase in CD103+CD8+ T_RM_ cells in tumor implants is unclear, but some CD103+CD8+ T_RM_ cells expressed Ki67, indicative of local proliferation (Figure 5C).

Although transferred RepTILs were not proficient in the control of the growth of tumor implants, the expression of granzyme B (GrB) by a few CD103+CD8+ T_RM_ cells demonstrates their tumor-killing potential (Figure 5D). Whereas trametinib treatment resulted in the highest density of CD103+CD8+ T_RM_ cells at the tumor margin for patient 16, the accumulation of these T_RM_ cells occurred both at the margin and within the tumor from patient 8 (Figure 5E). In general, the extent of effector responses by CD103+CD8+ T_RM_ cells, indicated by their expression of Ki67 (Figure 5F) and GrB (Figure 5G), followed the same trend as their repopulation of autologous tumor implants. Altogether, our data on the fate of transferred TILs suggest that a fraction of CD8+ TILs among in vitro–expanded RepTILs accumulate in various compartments of the autologous tumor as activated CD103+CD8+ T_RM_ cells.

### 2.8. Frequency of E-Cadherin+ PDX Tumor Cells Correlate with the Quantity of CD8+ T_RM_ Cells

**Figure 6:**
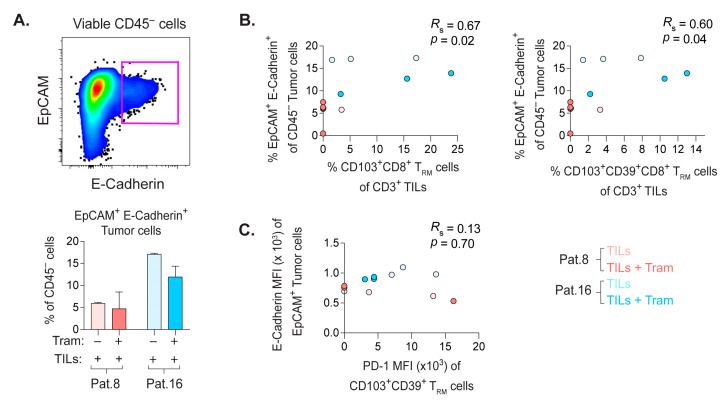
The frequency of E-Cadherin+ tumor cells associated with the percentage of CD8+ T_RM_ cells. (**A**) Gating of EpCAM+ E-Cadherin+ tumor cells from viable CD45– cells and their frequency in tumor implants of indicated groups of PDX mice. (**B**) Non-parametric Spearman correlation analyses of the frequencies of EpCAM+ E-Cadherin+ tumor cells vs. CD103+CD8+ T_RM_ cells or CD103+CD39+CD8+ T_RM_ cells. (**C**) Spearman correlation analysis of the MFI values of E-Cadherin on EpCAM+ tumor cells vs. PD-1 on CD103+CD39+CD8+ T_RM_ cells. Bars show mean with SD.

The proximity of CD8+T_RM_ cells to the tumor cells encouraged the characterization of markers expressed by the PDX tumor cells involved in the functional regulation (Supplementary Figure S5A–D) or intratumoral retention of TILs (Figure 6A–C).

The EpCAM+ tumor epithelial cells may, to some extent, compensate for the lack of human APC functions in the PDX model by expressing human leukocyte antigen (HLA)-ABC and HLA-DR (Supplementary Figure S5A,B). These HLA molecules were often higher on tumors of PDX mice receiving the transfer of RepTILs.

As the tumor environment is often immunosuppressive, we analyzed the expression of programmed death ligand-1 (PD-L1) on tumor cells that mediate T cell inhibition (Supplementary Figure S5C). The EpCAM+ cells of trametinib-treated PDX mice of patient 8 had slightly higher PD-L1 levels, but this marker was rather comparable between the groups. There was no correlation between the levels of PD-L1 on tumor cells and PD-1 on CD8+ T_RM_ cells co-expressing CD39, or not (Supplementary Figure S5D). Thus, the modest tumor control by RepTILs may not be primarily due to the PD-L1/PD-1 axis.

A large variety of human tumors express E-Cadherin (CD103 ligand) [28]. Binding of E-cadherin on PDX tumor cells to CD103+ CD8+ T_RM_ cells may sequester these TILs within the tumor implant, and the expression of E-Cadherin by EpCAM+ tumor cells was not affected by trametinib treatment (Figure 6A). Interestingly, the frequency of E-cadherin+ tumor cells correlated with the percentage of CD8+ T_RM_ cells co-expressing CD39, or not (Figure 6B). However, there was no link between the expression levels of E-Cadherin vs. PD-1 on CD103+CD39+CD8+ T_RM_ cells (Figure 6C).

Thus, the expression of the proliferation marker Ki67 on CD103+CD8+ T_RM_ cells (Figure 4C,F) and E-cadherin on tumor cells retaining T_RM_ cells may represent a few intratumoral events promoting the accumulation of CD103+CD8+ T_RM_ cells in CRLM-derived PDX tumors. However, the precise mechanisms behind the repopulation of T_RM_ cells in autologous tumors likely require additional in-depth transcriptomic studies.

## 3. Discussion

The consensus within the field of tumor immunology underpins the important role of TILs in CRC with regard to prognosis, post-treatment clinical outcome and treatment strategies [3,4,5,6,7]. Although functionally distinct TIL subsets exist in CRC [13,24,25,26], reports on TILs within CRLM are relatively scarce. Thus, we characterized T cell subsets in CRLM and the adjacent liver tissue.

Overall, the distinct microenvironments of CRLM and the adjacent liver were likely represented by increased frequencies of CD4+ αβ T cells in CRLM, combined with decreased levels of CD8+ αβ T cells and several unconventional T cell subsets. The elevated PD-1 expression on several unconventional TILs further confirms the distinct environment of CRLM, as well as their potential as immunotherapy targets. The contrasting milieus of these two tissues could also be depicted by the increased proportion of PDCs and more pronounced MDC activation in CRLM compared to the adjacent liver.

Our subsequent shift of focus towards T_RM_ cells within CRLM stems from recent reports proposing CD103+CD39+CD8+ TILs as tumor-reactive TILs in various tumors [11,12]. Whereas CD103+CD8+ T_RM_ cells were readily detected in both CRLM and the adjacent liver, T_RM_ cells that co-expressed CD39 were mainly present in CRLM. In line with our previous report on T_RM_ cells in primary CRC [13], the CD103+CD8+ T_RM_ cells in CRLM and the liver also had high expression of co-inhibitory markers, which indicate continuous T cell activation. In this regard, previous reports have identified CD39 as a marker of extended T cell activation [29] or tumor-specific TILs in CRC [12], which encouraged us to functionally compare CD103+CD8+ T_RM_ cells from CRLM vs. liver.

Surprisingly, CD103+CD8+ T_RM_ cells from both tissues that responded to cognate stimulation from peptides of microbial antigens also co-expressed CD39. Since CD103+CD8+ T_RM_ cells in the liver did not express CD39 ex vivo, this suggests that the increased frequency of CD103+CD39+CD8+ T_RM_ cells in the liver suspension after cognate antigen stimulation was due to CD39 upregulation during proliferation. As mentioned, the ex vivo frequency of CD103+CD39+CD8+ T_RM_ cells in CRLM was higher compared to in the liver, but whether these T_RM_ cells responding to cognate antigens upregulated or retained their CD39 expression remains unclear. Further, the co-residing APCs within CRLM vs. liver may differ in their capacity to provide persistent stimulation to T_RM_ cells and give rise to contrasting CD39 expression on T_RM_ cells in these two tissues.

The predominance of CD103+CD39+CD8+ T_RM_ cells in CRLM relative to the liver ex vivo and their response to cognate antigens in vitro encouraged us to assess their responses directed against the tumor rather than pathogen-based peptide antigens. We opted for an in vivo approach using a PDX model to assess tumor control by transferred TILs expanded from CRLM and the potential of trametinib to assist autologous anti-tumor responses. The opposing frequencies of CD4+ vs. CD8+ RepTILs between the two CRC patients, from which we succeeded in establishing PDX models, also provided the opportunity to associate tumor control with the specific ratio of TIL subsets.

Although RepTILs from both patients were capable of robust IFN-γ responses in vitro, their control of large or small CRLM-derived tumor implants was overall subpar in vivo. As the PDX mice lack patient-derived APCs, the inefficient tumor control of RepTILs may relate to inadequate tumor antigen presentation and stimulation. Moreover, most CRLMs present intact DNA mismatch repair ability [30,31], giving rise to microsatellite-stable (MSS) tumors with a low mutation load. The MSS tumors are therefore less immunogenic and likely to mount a limited number and variety of tumor-reactive TILs, as well as generate insufficient proportions of tumor-specific CD8+ T_RM_ cells. Surprisingly, the transfer of RepTILs with a high frequency of CD4+ TILs and subsequent trametinib treatment induced modest, albeit persistent, control of smaller tumor implants. In contrast, the transfer of RepTILs containing more CD8+TILs mediated the transient control of smaller tumor implants during trametinib treatment. It is plausible that MEKi, together with the abundance of CD4+ TILs, favors immunological pathways for qualitatively better tumor control. In addition, the frequency of immunosuppressive Tregs among CD4+ TILs could be central in the prognosis and/or the choice of treatment, since Tregs abundantly express markers targeted by immune checkpoint inhibition therapy [32]. Overall, trametinib treatment and RepTILs from the two patients with distinct CD4 vs. CD8 TIL ratios induced momentary or stagnant control of smaller tumors.

Several mouse tumor models have demonstrated robust tumor rejection along with an elevated frequency of CD8+ TILs after treatment composed of trametinib combined with other therapeutic drugs [17,18,19,20,21]. However, these murine studies did not address the increase in mouse T_RM_ cells or whether the rise in CD8+ TIL numbers reflects general or tumor-restricted T cell elevation. We quantified CD103+CD8+ T_RM_ cells in the spleen, blood and tumor tissue and found an increase in these T_RM_ cells strictly in the autologous CRLM-derived tumor implants. It is plausible that the restricted increase in T_RM_ cells in tumor implants represents an intrinsic preference of these TILs to revisit the autologous CRLM tissue that they were isolated from. However, the expression of the proliferation marker Ki67 may suggest intratumoral expansion, and E-Cadherin expressed by tumor cells may retain CD8+ T_RM_ cells within the tumor implant upon binding to CD103.

In our PDX model, trametinib induced a more evident fold increase in CD103+CD8+ T_RM_ cells in tumor implants of PDX mice that received RepTILs with high CD4+ TIL numbers, and the transfer of RepTILs rich in CD8+ TILs encompassed an increase in intratumoral T_RM_ cells regardless of trametinib. To this end, the CD103+CD8+ T_RM_ cells detected at the tumor stroma and margin, as well as within the tumor epithelia, expressed markers of tumor-killing activity, which may represent anti-tumor responses of T_RM_ cells targeting autologous CRLM-derived tumors with unsurmountable growth capacity.

## 4. Materials and Methods

### 4.1. CRC Patients

Signed informed consent was obtained from all patients included in the study. The patients presenting CRLMs underwent liver surgery at the Sahlgrenska University Hospital. The majority of patients presented synchronous tumors, and additional patient data are provided in Supplementary Table S1. Metastatic tumor tissue closest to the tumor border and macroscopically tumor-free liver tissue (around 5 cm away from the tumor border) were collected from the liver resectate. The tissues were transported in RPMI 1640 (Gibco, Thermo Fisher, Carlsbad, CA, USA) on ice for generation of single-cell suspensions within 30 min. For some patients, 5 × 5 mm tumor pieces were used for the establishment of the PDX model and in vitro expansion of TILs [23].

### 4.2. Single-Cell Suspensions from Patient Samples

Tumor and liver tissues were cut into 3 × 3 mm pieces with scissors and enzymatically digested at +37 °C with 25 μg/mL Liberase TM (Roche, Basel, Switzerland) and 20 μg/mL DNase I (Sigma, St. Louis, MO, USA) in RPMI 1640. After 1 h digestion, enzyme activity was quenched by the addition of 1 mL of complete medium consisting of RPMI 1640 with 10% FCS, 1% Penicillin/Streptomycin, 1% Hepes buffer and 0.1% Gentamycin (Gibco). Subsequently, the obtained cell suspensions were filtered through a 70 μm cell strainer (BD, San Jose, CA, USA) and washed with PBS. The pelleted cells were resuspended to 1 × 10^6^ cells/mL and used for ex vivo characterization and in vitro assays.

### 4.3. Flow Cytometry

Cell suspensions were stained as previously described [13,24,27]. Briefly, cell viability was determined by Zombie Red Fixable viability dye (Biolegend, San Diego, CA, USA). Subsequently, cells were incubated with fluorescent antibodies (Supplementary Table S2) for 20 min at room temperature, washed with PBS and fixed with 2% paraformaldehyde. Intracellular staining of IFN-γ was performed using Fixation/Permeabilization Solution Kit (BD) as previously described [24]. Samples were acquired using a BD LSRFortessa flow cytometer and analyzed with FlowJo v.9.9.6 (Tree Star, Ashland, OR, USA).

### 4.4. T Cell Proliferation Assay

CRLM and liver suspensions were resuspended with 5 mL of PBS and overlayed on a Ficoll gradient (GE Healthcare, Uppsala, Sweden). After 20 min of centrifugation at 870× *g* without brake and with the lowest acceleration, the interphase enriched in leukocytes was collected, washed and labeled with 1μM Carboxyfluorescein succinimidyl ester (CFSE) (Molecular Probes, Eugene, OR, USA) in 1 × 10^6^ cells/mL concentration for 7 min at +37 °C. Labeling was quenched with 1mL of FCS, followed by a PBS wash. CFSE-labeled cells were resuspended to 1 × 10^6^ cells/mL with complete media and cultured for 4 days with and without 2 μg of pooled peptides based on antigens from Cytomegalovirus, Epstein–Barr virus, Flu (influenza) virus, plus Tetanus toxoid (PepMix CEFT pool) (JPT Peptide Technologies, Berlin, Germany), or 2 μL of MACSiBead particles loaded with anti- CD2, CD3 and CD28 antibodies (Miltenyi Biotec, Auburn, CA, USA). Proliferating T cells, indicated by their dilution of CFSE dye, were assessed by flow cytometry.

### 4.5. Expansion and Stimulation of TILs In Vitro

Tumor pieces (approximately 1 × 1 mm) were placed in 24-well plates (Nunc, Thermo Fisher) and submerged in 1 mL of human IL-2 (hIL-2) medium, consisting of RPMI 1640 with 6000 IU/mL recombinant human IL-2 (PeproTech, East Windsor, NJ, USA), 10% heat-inactivated human AB serum (Sigma) and 50 μg/mL Gentamicin (Gibco). TILs that emigrated from the tumor pieces and subsequently expanded in response to hIL-2 are referred to as young-TILs. Every second day, 0.5 mL of medium was replaced with fresh hIL-2 medium. For the uniform expansion rate in all wells, 100 μL cell suspensions from wells with visibly more young-TILs were transferred to wells with fewer cells. After 20–30 days of expansion, the yield of young-TILs was typically 50–90 ×10^6^, which were then cryopreserved. For the transfer of 10 × 10^6^ RepTILs/PDX mouse, the cryostored young-TILs were thawed, rested overnight in hIL-2 medium and further expanded with the rapid expansion protocol [23]. Briefly, 1 × 10^5^ young-TILs in a 25 cm^2^ culture flask (Sarstedt) were added to 2 × 10^7^ irradiated feeder cells (allogeneic PBMCs exposed to 40 Gy), 30 ng/mL anti-CD3 antibody (clone: OKT3, Miltenyi Biotec), 10 mL of hIL-2 medium and 10 mL of REP medium, which consisted of AIM-V (Invitrogen, Thermo Fisher) with 10% human serum and 6000 IU/mL hIL-2. Flasks were incubated in an upright position at +37 °C. On day 5, half of the REP medium was replaced. On day 7 and every subsequent day, RepTILs were split into additional flasks and adjusted to 1–2 × 10^6^ cells/mL/flask with REP medium. After 10–14 days, RepTILs were harvested and used for experiments. RepTILs were cultured for 6 h with or without 2 μL of MACSiBead particles, and IFN-γ staining was performed as previously described [24,27].

### 4.6. Establishment of PDX Mouse Model and Treatments

The design and performance of animal experiments in the current study were in accordance with EU Directive 2010/63. PDX mice (PDXv.2 model, [23]), and were established as follows: small tumor pieces were placed under the skin on the flank of 6–15-week-old NOG mice (non-obese severe combined immune-deficient interleukin-2 chain receptor γ knockout mice, Taconic, Denmark). Tumors were measured weekly with calipers, and tumor volumes were calculated as width × width × length/2. For further transplantations or treatments, tumors were extracted, and tumor suspensions were serially passaged via subcutaneous injections to NOG mice or human IL-2 transgenic NOG (hIL2-NOG) mice (Taconic). For TIL treatments, 10 × 10^6^ RepTILs were transferred intravenously to hIL-2 NOG mice with actively growing tumors, as confirmed by caliper measurements. Trametinib was mixed into the chow at 2.5 mg/kg, resulting in an approximate dose of 0.5 mg/kg mouse per day. Trametinib treatment started around 3 weeks after RepTIL transfer.

### 4.7. Samples from PDX Model

Mice carrying patient-derived tumors were sacrificed before or when tumor implants had reached the ethical size limit. The implanted tumors, spleens and whole blood were collected from the sacrificed mice. Single-cell suspensions of tumor implants were obtained using the same protocol as described above. Spleens were cut into 3 × 3 mm pieces that were passed through a 70 μm cell strainer (BD) with a syringe plunger, and the obtained cell suspensions were washed with PBS. Blood was collected into Microvette EDTA tubes (Sarstedt, Nümbrecht, Germany) from the hind leg vein (vena saphena) and centrifuged for 5 min at 2000× *g*. Subsequently, the plasma was removed and replaced with PBS prior to PBMC isolation with a 2 mL Ficoll gradient in 5 mL polystyrene tubes (Corning, Amsterdam, The Netherlands). Sanger sequencing for *KRAS* status was outsourced to Eurofins Genomics (Ebersberg, Germany).

### 4.8. Immunohistochemistry

Antigen retrieval was performed on 6 μm thick sections of formalin-fixed paraffin-embedded implanted tumors on positively charged slides using IHC antigen retrieval solution–High pH (Invitrogen, Thermo Fisher) and 2100 Retriever pressure cooker (Aptum Biologics, Southampton, UK). Two rounds of pressure cooking were applied, and slides were cooled off while remaining in the pressure cooker. Moisture around the sections was wiped off with tissues before encircling the sections with a hydrophobic pen (Histolab, Gothenburg, Sweden). The antigen markers (Supplementary Table S3) were sequentially stained at room temperature. Staining of each marker started with 10 min in blocking buffer consisting of 0.1% bovine serum albumin (Thermo Fisher) in PBS. Sections were then incubated with primary antibodies for 50 min, washed twice with PBS (3 min/wash), incubated with horseradish peroxidase-conjugated secondary reagents for 10 min, washed again with PBS and, finally, incubated with tyramide dye (HRP substrate) for 10 min per the manufacturer’s protocol. Subsequently, primary and secondary antibodies were removed from the sections by submerging the slides in IHC antigen retrieval solution–High pH, followed by heating in a microwave oven at 700 W for 1 min at 70 W for 10 min. Slides were cooled down and washed with PBS prior to staining of the next marker. When all markers were stained, cell nuclei were labeled with 4′, 6-diamidino-2-phenylindole (DAPI) (Sigma) for 10 min, washed with PBS and mounted with Prolong Gold Antifade media (Molecular Probes).

### 4.9. Image Analyses

Slides were scanned with TissueFAXS CHROMA (TissueGnostics, Vienna, Austria) equipped with 7-channel SpectraSplit filter sets (Kromnigon, Gothenburg, Sweden) and a Hamamatsu C13440-20CU digital camera (Hamamatsu Photonics, Hamamatsu, Japan). Automated scanning of entire tissue was performed at 20× magnification and acquired z-stacks from each fluorescence channel on any field consisting of images from one focus plane, plus three positions above and three below (7 images/channel/field). Scanned images were analyzed with StrataQuest (v. 7.0.1.189; TissueGnostics). Individual cells were distinguished by DAPI staining, and their phenotype was determined by intensities of the stained markers. The machine learning process developed through our collaboration with TissueGnostics, enabled the software to separate tumor epithelia and stroma, as well as segmentation of stromal areas according to specified distances relative to the tumor bed.

### 4.10. Statistical Analyses

Paired (Wilcoxon signed-rank test) and unpaired (Mann–Whitney test) comparisons, plus Spearman correlation analyses were performed using GraphPad Prism software, v.9.0.2. (San Diego, CA, USA), and considered significant at *p* < 0.05.

## 5. Conclusions

Although the establishment of the PDX model from resected CRLM can be challenging, this in vivo model of TIL responses against autologous CRLM has the ability to report a wide range of anti-tumor responses in treated CRC patients, including persistent or transient tumor control. Importantly, our in vivo data support the emergence of intratumoral CD103+CD8+ T_RM_ cells as targets for T-cell-mediated anti-tumor therapy. Of note, the reappearance of activated T_RM_ cells strictly within the autologous tumor is likely desirable, since this enables them to exert effector responses within the tumor rather than off-target tissues, thereby minimizing therapy-related adverse events.

## Data Availability

Data are contained within the article and Supplementary Materials.

## Supplementary Materials

The following supporting information can be downloaded at: https://www.mdpi.com/article/10.3390/cancers14122882/s1. Figure S1: Characterization of APCs in CRLM and adjacent liver tissue. Figure S2: Modest improvement in growth control of tumor implants of limited size by autologous RepTILs in trametinib-treated PDX mice. Figure S3: Memory CD8 T cells in tumor implants contain CD103+ T_RM_ cells and CD103− TILs. Figure S4: Frequency of Tregs in tumor implants of PDX mice. Figure S5: Characterization of EpCAM+ cells of tumor implants. Table S1: Characteristics of colorectal cancer patients with liver metastases, Table S2: Fluorescence-conjugated antibodies for flow cytometry. Table S3: Reagents for in situ staining of PDX tumor sections.
