# Supplementary material for: A Fraction of CD8+ T Cells from Colorectal Liver Metastases Preferentially Repopulate Autologous Patient-Derived Xenograft Tumors as Tissue-Resident Memory T Cells
Source: Cancers (Basel). 2022 Jun 10;14(12):2882. doi: 10.3390/cancers14122882 (PMC9221137; doi:10.3390/cancers14122882)
Supplement: Supplementary file 1 [file cancers-14-02882-s001.zip › cancers-1729421-supplementary.pdf]

Supplementary Materials

Table S1. Characteristics of colorectal cancer patients with liver metastases.

		Females	Males
	n (15)	5	10
	Age range	58–71	44–78
	Age median	67	62
	Synchronous tumor	4	7
	Metachronous tumor	1	3
Primary tumor location	Right colon	0	2
	Left colon	5	3
	Rectum	0	5
	CEA (ng/mL)	1–25	1–8
	Median CEA (ng/mL)	7.5	8.8
	Untreated	1	7
Neoadjuvant	Capecitabine/Oxaliplatin	0	2
	Leucovorin/Fluorouracil/ Oxaliplatin	4	1

Table S2. Fluorescence-conjugated antibodies for flow cytometry.

Antibody	Clone	Manufacturer
CD3	UCHT1	BD Biosciences
CD4	OKT4	Biolegend
CD8	RPA-T8	BD Biosciences
CD14	M5E2	Biolegend
CD16	3G8	Biolegend
CD19	SJ25C1	BD Biosciences
CD25	2A3	BD Biosciences
CD39	A1	Biolegend
CD56	HCD56	Biolegend
CD80	L307.4	BD Biosciences
CD1c	L161	Biolegend
CD11c	B-ly6	BD Biosciences
CD45	HI30	BD Biosciences
CD45RO	UCHL1	BD Biosciences
CD103	Ber-ACT8	BD Biosciences
CD123	9F5	BD Biosciences
CD127	A019D5	Biolegend
CD141	1A4	BD Biosciences
CD163	RM3/1	Biolegend
HLA-ABC	G46-2.6	BD Biosciences
HLA-DR	G46-6	BD Biosciences
TCR γδ	B1	BD Biosciences
TCR Vα7.2	3C10	Biolegend
PD-1	EH12.1	BD Biosciences
LAG-3	11C3C65	Biolegend
TIM-3	7D3	BD Biosciences
PD-L1	29E.2A3	Biolegend
EpCAM	9C4	Biolegend
E-Cadherin	67A4	Biolegend
IFN-γ	B27	BD Biosciences

Table S3. Reagents for in situ staining of PDX tumor sections

Primary antibodies	Clone	Vendor
CD3	Polyclonal	Dako, Glostrup, Denmark
CD8	SP16	Invitrogen, Waltham, MA, USA
CD103	EPR466-(2)	Abcam, Cambridge, UK
Granzyme B	EPR22645-206	Abcam
Ki67	MIB-1	Dako
Pan-Cytokeratin	KRT/1877R	Abcam
**HRP substrates**	**Marker visualized**	**Vendor**
CF430	CD3	Biotium, Fremont, CA, USA
CF488	Ki67	Biotium
CF594	CD8	Biotium
TSA-DIG & Opal Polaris 780	Granzyme B	Akoya, Marlborough, MA, USA
TSA Cyanine 3	CD103	Akoya
TSA Cyanine 5	Pan-Cytokeratin	Akoya

Horseradish peroxidase (HRP)-conjugated secondary antibodies: Envision FLEX/HRP (Dako), or Opal Polymer HRP Ms+Rb (Akoya). Cell nuclei were stained with DAPI (Sigma, St. Louis, MO, USA).

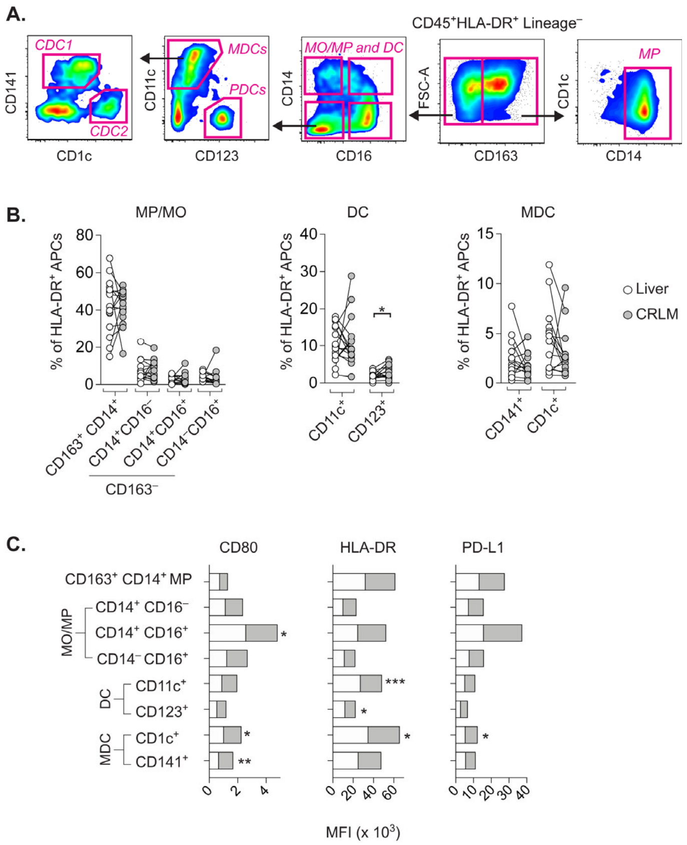

Figure S1. Characterization of APCs in CRLM and adjacent liver tissue. (**A**) Gating strategy of APC subsets, as shown with single cell suspension of CRLM. Viable, HLA-DR+ and lineage (CD3, CD19, CD56)– cells of CD45+ leukocytes were separated into CD163– and CD163+ fractions. The CD14+ macrophages (MPs) were gated from CD163+ cells. The CD163– fraction contained subsets of monocytes (MOs) and/or MPs that were defined by their CD14 and CD16 expression. The CD163–CD14–CD16– dendritic cells (DCs) comprised CD123+ plasmacytoid DCs (PDCs) and CD11c+ myeloid DCs (MDCs). The MDCs were further separated into subsets known as, CD141+ CDC1 and CD1c+ CDC2. (**B**) Frequency of indicated APC subsets among total HLA-DR+ APCs in CRLM vs. adjacent liver tissue. (**C**) Stacked bars show the proportions of MFI on the staining of CD80, HLA-DR and PD-L1 on APC subsets in CRLM and liver, respectively. (*p <0.05, **p <0.01, ***p <0.001, Wilcoxon test).

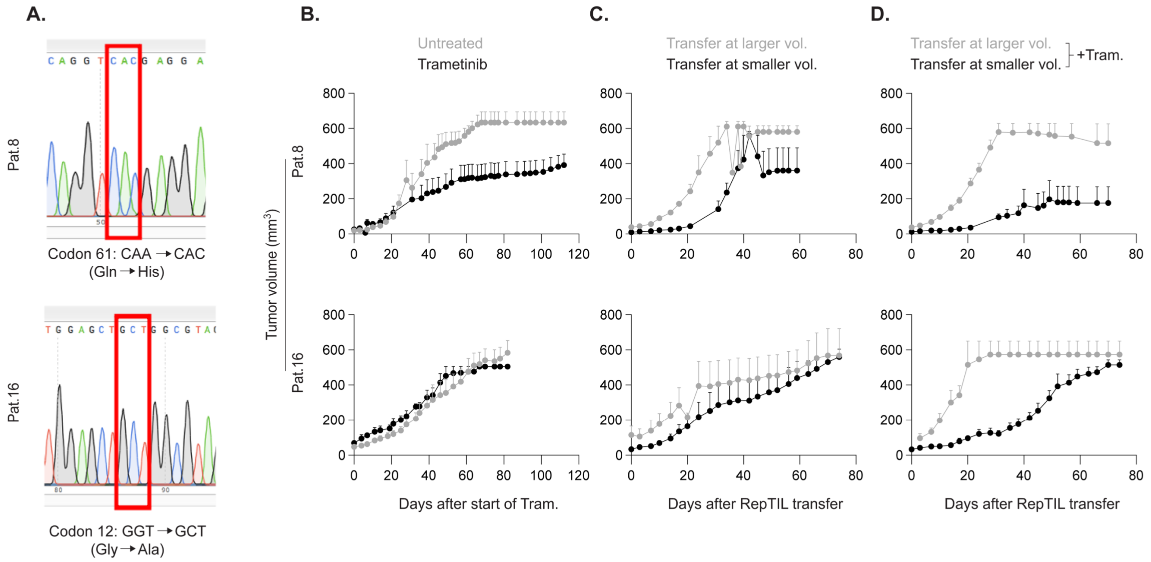

Figure S2. Modest improvement in growth control of tumor implants of limited size by autologous RepTILs in trametinib-treated PDX mice. The PDX mice with subcutaneous tumor implants derived from CRLM of patient 8, or patient 16, were separated into groups that received transfer of RepTILs, or not. Both groups were further divided into groups that received trametinib, or left untreated. (**A**) Sanger sequencing of mutations in the *KRAS* gene in CRLM samples. Red boxes show altered nucleotide sequence in codon 61 (patient 8) and codon 12 (patient 16), respectively. (**B**) Cumulative volumes of tumors derived from patient 8 or 16, in untreated and trametinib-treated PDX mice that did not receive transfer of RepTILs. (**C**) Cumulative tumor volumes in untreated PDX mice that received RepTIL transfer when tumor implants were above or below a specific volume (20 mm^3 and 50 mm^3 for patient 8 and 16, respectively). (**D**) Cumulative tumor volumes in PDX mice that received RepTIL transfer at the same time as PDX mice in C, and then treated with trametinib around 3 weeks later.

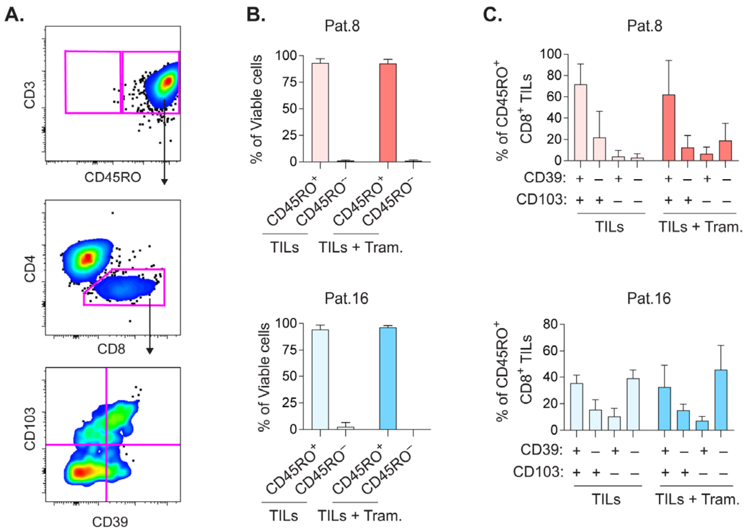

Figure S3. Memory CD8 T cells in tumor implants contain CD103+ T_{RM} cells and CD103– TILs. (**A**) Gating strategy of CD45RO+ memory CD8 T cell subsets defined by CD103 and CD39 expression. (**B**) Compiled data from 3 PDX mice/patient, with regards to percentage of CD3+CD45RO+ memory T cells among live cells in the tumor implants of PDX mice representing patient 8 or 16, which received RepTILs with or without subsequent trametinib treatment. (**C**) Frequency of CD103+CD39+ T_{RM} cells, CD103+CD39– T_{RM} cells, CD103–CD39+ T cells, or CD103–CD39– T cells within CD45RO+CD8+ memory T cells in tumor explants of indicated groups. Bars show the mean with SD.

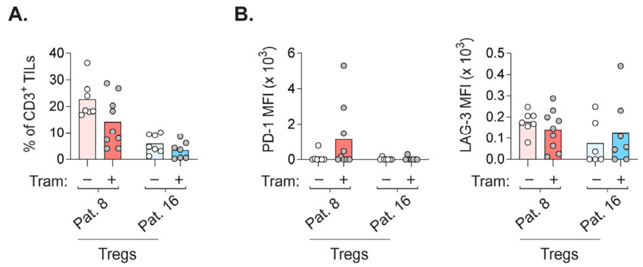

Figure S4. Frequency of Tregs in tumor implants of PDX mice. The CD4+CD127 dim/–CD25+ Tregs were identified using the same gating strategy as shown in Figure 1. (**A**) Frequency of in tumor implants from the indicated groups of PDX mice representing patient 8 and 16, respectively. (**B**) Expression of PD-1 and LAG-3 by the Tregs. Bars show the mean.

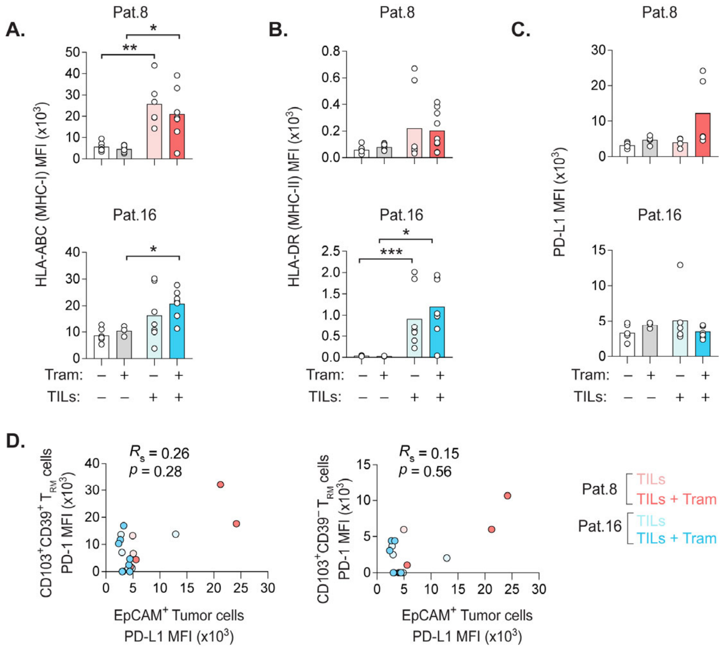

Figure S5. Characterization of EpCAM+ cells of tumor implants. The PDX mice with tumor implants from patients 8 or 16, were treated as described in Figure S2. The MFI on staining of HLA-ABC (MHC-I) (**A**), HLA-DR (MHC-II) (**B**), and PD-L1 (**C**) on EpCAM+ tumor epithelial cells from tumor implants of the specified groups. (**D**) Non-parametric Spearman correlation analyses of PD-1 MFI of CD103+CD39+CD8+ T$_{RM}$ cells, or CD103+CD39–CD8+ T$_{RM}$ cells vs. PD-L1 MFI of EpCAM+ tumor cells. Bars show the mean.
